# Supplementary material for: Deep time evolution of the Latitudinal Diversity Gradient: Insights from mechanistic models
Source: PLoS One. 2025 Sep 25;20(9):e0332766. doi: 10.1371/journal.pone.0332766 (PMC12463215; doi:10.1371/journal.pone.0332766)
Supplement: Attachment — Submitted filename: Comments2authors.pdf [file pone.0332766.s005.pdf]

This manuscript presents a simulation-based investigation into the origins and temporal dynamics of the Latitudinal Diversity Gradient (LDG) in terrestrial mammals. Using mechanistic models informed by fossil and phylogenetic data, the authors simulate biodiversity patterns from the Cretaceous to the present. The models reveal the emergence and steepening of the LDG beginning around the Late Cretaceous to the early Paleogene (~50–35 Ma), shaped by tropical speciation, poleward dispersal, extinction gradients, and geographic asymmetries. The findings are generally consistent with the "Out of the Tropics" hypothesis, and emphasize the roles of both abiotic and biotic drivers in shaping global biodiversity patterns.

The study is highly ambitious in scope and tackles a long-standing question in macroecology: the emergence and persistence of the LDG. The integration of simulations with fossil and phylogenetic evidence is commendable, as is the emphasis on deep time dynamics. However, several points require clarification or expansion to strengthen the manuscript's impact and clarity:

- The paper lacks a clear conceptual framework. The introduction could better define key terms (e.g., LDG, diversification vs. speciation), summarize aims more explicitly, and structure the various hypotheses and drivers in a more organized way (e.g., abiotic vs. biotic, temporal vs. spatial). The current structure feels list-like and sometimes inconsistent between sections (e.g., intro vs. discussion).

- While the modeling framework is rich, the paper remains overly descriptive in presenting results and does not fully exploit the model's potential to test mechanisms or disentangle the roles of different processes. Several general and specific comments request deeper comparative analysis between model scenarios and more explicit discussion of the model's assumptions, simplifications (e.g., speciation), and limitations.

- The writing is often dense, technical, or ambiguous, especially in methodological sections. Key processes (e.g., dispersal, turnover, ecological dynamics) are introduced with vague or overly complex language, and some terms (e.g., "surface processes", "cost function") are used without clarification. Readers unfamiliar with grid-based simulation models may struggle to follow the logic without additional explanations, definitions, or simplification.

- The paper sometimes fails to tie together its aims, methods, and interpretations. For example, some ideas appear in the discussion without being developed earlier, and the conclusion repeats previous points without offering a strong synthesis. The discussion does not fully explain why certain patterns (e.g., turnover asymmetry, cradle/museum dynamics) arise, and conservation implications remain underdeveloped despite being potentially important.

Below you will find more general and specific comments associated with specific line numbers in the ms.

General comments

1. **8-11:** The reader may want to know how these biotic factors relate or differ from common ideas about "time for speciation", "speciation rates", and "ecological limits".
2. **13-19:** Same general comment as above, i.e. can these multiple factors be organized in some way that makes it less like a list and more like a categorization of different temporal, ecological, and evolutionary effects?
3. **22-26:** Here the authors seem to try to connect the list of factors above with specific hypotheses that are based on speciation, migration, and diversification. However, the link is not clear. A better structuring of the text and maybe even a conceptual figure would help.
4. **67-76:** It seems like the authors are trying to specify their modeling scope in this last paragraph but it is not 100% clear. I suggest being even more clear on exactly what processes and mechanisms are modeled and why. Also, they may consider merging this paragraph with the preceding one as I think it would improve the flow of the introduction.
5. **111-115:** Here the authors probably want to do a better job describing exactly what the biodiversity processes and mechanisms are. Maybe a conceptual figure or a table would help.
6. **117:** It is unclear and a bit confusing to the reader, as it is not clear if these model specifics are included in all the scenarios mentioned above (also the ones that are "solely based on climate and tectonics").
7. **121-130:** The way dispersal is framed as some cost is not intuitive to me as a first-time reader. I was hoping that the authors can frame this in a more intuitive way with more focus on actual dispersal and less on costs.
8. **132-136:** I am sure the authors realize that this is an extremely simplified view of speciation. This is of course fine in a modelling study but the authors need to discuss the potential consequences of such simplifications for the results.
9. **138-147**: This section is described as if it was a Ornstin-Uhlenbeck process but it is really not as OU processes are commonly described as a continuous model. At the same time the authors mix in concepts like Brownian motion which is commonly completely undirected. This is confusing. I suggest that the authors revise this part such that it goes more in line with the standard terminology of directed stochastic models.
10. **226-239:** For someone that is not an expert in these types of grid-based models this section becomes very dense. I suggest the authors try to revise this such that it becomes more accessible to non-experts.
11. **253-261:** Seems more appropriate to present this in the methods section. A reminder here in the results may be justified but should also be clearly described in the methods section.
12. **272-276:** I may have missed somthing but I think that the authors fall short in actually "Understanding the LDG". Rather than concluding that biodiversity is correlated with precipitation (which is a trivial observation) they can actually disentangle some of the more interesting explanations that are based on dispersal, ecology, adaptation, and speciation. I thus call for a more indepth analyses of the different model scenarios Table 1 and how they preform and why.

13. **293:** General: This whole section is very descriptive with very little explanations of why these patterns emerge. Given that the authors run different models (Table 1) they should be able to provide insights to the underpinnings of the observed patterns.
14. **376-377:** This may be the most concerning result that comes with this study — the fact that different model scenarios give very similar outputs. It essentially means that the authors have limited information from the models that can help them disentangle the relative importance of different factors affecting the biodiversity patterns. The major goal of this paper thus seems to be out of scope for the particular model and model scenarios that were chosen by the authors.
15. **431:** In this section the authors provide some detail on potential drivers of biodiversity, but they are all more or less abiotic or environmental effects. Such explanations are quite different from the focus that was introduced in the introduction and early parts of the model presentation, i.e. dispersal, ecology, adaptation, and speciation. Thus it seems to me like the authors are missing their own mark that was set up in the introduction and early parts of the model description.
16. **522:** Quite wordy section that does not provide a lot of insight. Repeating some of the key results and telling the reader that similar (or dissimilar)...
17. **568:** Although the discussion about cradle and museum is interesting, I cannot help but ask the "why" question. I was hoping for more answers along the lines of the questions asked in the introduction of this paper.
18. **621:** In this section the authors are scratching the surface of what I am calling for above. I suggest the authors try to have such analyses of the comparison between model scenarios permeate the paper much better. This is where I see the real contribution from this modeling study.
19. **699:** This conclusions section does not bring much new or outstanding information. I suspect that this whole section can be deleted without loss of readability or clarity — given that the other parts of the discussion are tightened, that is.

Specific Comments

1. Watch out for long sentences in abstract. Also, the abstract could benefit from ending with an outlook and future perspectives sentence.
2. **21-22:** Definitions and distinctions between diversification and speciation probably needed here.
3. **49-50:** A few follow-up sentences that provide explicit examples of mechanisms that have been modeled, and specific modeling studies and their results, may be good to add here, as it would help the reader understand what is meant and what possibilities these types of models provide.
4. **65:** Even though the authors list key features of the model above, it may be worth being very clear — i.e., reminding the reader about what mechanisms they are focused on in this study.
5. **81:** “These dynamics” ,unclear — be specific.
6. **86-87:** Not sure what this sentence is trying to say. I suggest either clarifying how the Game of Life model has inspired Gen3sis or deleting the sentence altogether.
7. **93:** Not sure what this means — please clarify.
8. **93:** The reader is kept in the dark about what these biological functions and behavioral laws may be. I suggest that the authors provide a few examples here to relieve the reader’s frustration a bit.
9. **121:** Please specify the temporal extent of such a time step.

10. **121:** Terminology used here implies that the reader should know what a "cost function" is and how it connects to dispersal. This is, however, not clear to me.
11. **132-136:** As I am sure the authors realize, this is an extremely simplified view of speciation. This is of course fine in a modeling study like this, but the authors need to discuss the potential consequences of such simplifications for the results.
12. **138-147:** This section is described as if it were an Ornstein-Uhlenbeck process, but it is really not, as OU processes are commonly described as continuous models. At the same time, the authors mix in concepts like Brownian motion, which is commonly completely undirected. This is confusing. I suggest that the authors revise this part such that it aligns more closely with standard terminology for directed stochastic models.
13. **176-177:** It may be worth describing why such an approach is conducted — i.e., a follow-up sentence describing how these 100 simulations were treated in downstream analyses.
14. **179:** These 1 Myr time steps are huge in the context of dispersal and ecology. I strongly suggest that the authors discuss what consequences such long time steps, combined with the fact that different modeled processes act on very different time scales, have for the model behavior and output.
15. **194-194:** Should this not be part of the model specifics on dispersal presented above?
16. **199:** See comment above about OU process.
17. **221-224:** Seems more like a discussion point than a statement in the methods section.
18. Figure 4 would benefit from panel titles telling the reader which panel is showing which hemisphere results.
19. **282:** The authors fall short in justifying this claim. See my general call for in-depth analyses of the different model scenarios and how they perform — and why.
20. **284-285:** Not sure this is suitable for the results section — rather part of a discussion.
21. **519-520:** Yes, I agree that this is what the model can do and should do. But unfortunately, I do not think the authors utilize their model to its full potential. Rather than explaining the mechanisms underpinning LDGs, they resort to a very descriptive presentation of their results that essentially boils down to environmental and/or abiotic factors — despite the fact that they frame their study from several biotic perspectives in the introduction.
