# Supplementary material for: Deep time evolution of the Latitudinal Diversity Gradient: Insights from mechanistic models
Source: PLoS One. 2025 Sep 25;20(9):e0332766. doi: 10.1371/journal.pone.0332766 (PMC12463215; doi:10.1371/journal.pone.0332766)
Supplement: Attachment — Submitted filename: Response to Reviewers.pdf [file pone.0332766.s006.pdf]

Dear Dr Kiel,

We are grateful for taking the time to provide feedback and useful comments on our manuscript. We are glad that you find our work interesting and potentially suitable for publication in PLoS One. We have carefully addressed your concerns and those rasied by our reviewers with great interest and care, and have significantly updated the manuscript. Our responses to the reviewers' comments are listed below in blue. Notably we have:

1) Made major changes to the structure of the text to streamline the manuscript, removing the focus on a narrow set of hypotheses and focusing more broadly on the fundamental ways paleoenvironmental change shape biodiversity gradients impacting speciation, extinction and dispersal.

2) Include more methodological detail to help readers better understand the model

3) Made the result and discussion sections more concise by reducing wordy descriptions that were diluting the main findings with too much information.

We have created a new version of the manuscript in PDF format as well as a PDF file with the differences and corrections from the first version. We hope that our revised manuscript will now reach the level of quality required for the PLoS One journal.

Thank you for your time and consideration.

Manon Lorcery and co-authors

Main comments

- The paper lacks a clear conceptual framework. The introduction could better define key terms (e.g., LDG, diversification vs. speciation), summarize aims more explicitly, and structure the various hypotheses and drivers in a more organized way (e.g., abiotic vs. biotic, temporal vs. spatial). The current structure feels list-like and sometimes inconsistent between sections (e.g., intro vs. Discussion).

We acknowledge these comments and we modified the introduction accordingly, by narrowing the focus on a set of hypotheses and focusing more broadly on the fundamental ways paleoenvironmental change shape biodiversity gradients through the effect on speciation, extinction and dispersal. These changes make the introduction more consistent with the discussion.

- While the modeling framework is rich, the paper remains overly descriptive in presenting results and does not fully exploit the model's potential to test mechanisms or disentangle the roles of different processes. Several general and specific comments request deeper comparative analysis between model scenarios and more explicit discussion of the model's assumptions, simplifications (e.g., speciation), and limitations.

We agree and made the results more concise by reducing descriptions that were diluting the main findings. In this revised version, we explained more clearly the biotic and abiotic mechanisms underlying the biodiversity trends highlighted by the models. These revisions overall help the paper better align with the scope stated in the introduction.

- The writing is often dense, technical, or ambiguous, especially in methodological sections. Key processes (e.g., dispersal, turnover, ecological dynamics) are introduced with vague or overly complex language, and some terms (e.g., "surface processes","cost function") are used without clarification. Readers unfamiliar with grid-based simulation models may struggle to follow the logic without additional explanations, definitions, or simplification.

We agree, the methodology could have benefited from more explanations and examples for a better understanding of the modeling framework. In this revised version, we provide clearer explanations of concepts and keys processes, and we also grouped model specifics with parameters to improve both clarity and the logical flow of the methodology–since parameter ranges are shared between the different scenarios.

- The paper sometimes fails to tie together its aims, methods, and interpretations. For example, some ideas appear in the discussion without being developed earlier, and the conclusion repeats previous points without offering a strong synthesis. The discussion does not fully explain why certain patterns (e.g., turnover asymmetry, cradle/museum dynamics) arise, and conservation implications remain underdeveloped despite being potentially important.

We agree and modified the paper to better tie its scope from the introduction to the conclusion. We have shortened the conclusion and restate only key results from this study. We also examined in greater details biotic and abiotic interlinks and how these patterns emerge in the result and discussion. Conservation implications, although marginally related to the scope of this article, have been better stated in this new version, highlighting the need to understand the stability of tropical ecosystems in order to safeguard them.

Reviewer 1 - General comments

1. 8-11: The reader may want to know how these biotic factors relate or differ from common ideas about "time for speciation", "speciation rates", and "ecological limits".

This is a great suggestion, thank you. The introduction in the first version was confusing, so we revised it to better narrow the focus of the paper and link it more clearly to our observations and discussion. In this revision, we removed the paragraph listing broad biotic factors, instead stating that there are countless hypotheses for the LDG, differing in the relative importance they give to different ecological, evolutionary or temporal factors (Lines 7-9)

2. 13-19: Same general comment as above, i.e. can these multiple factors be organized in some way that makes it less like a list and more like a categorization of different temporal, ecological, and evolutionary effects?

Following a similar structure as above, we briefly state that these numerous hypotheses differ in the relative importance they give to different ecological, evolutionary or temporal factors while having a shared foundation based on how variation in speciation, extinction and dispersal manifests across landscapes over time (Lines 9-11).

3. 22-26: Here the authors seem to try to connect the list of factors above with specific hypotheses that are based on speciation, migration, and diversification. However, the link is not clear. A better structuring of the text and maybe even a conceptual figure would help.

We recognize that the link we initially made was unclear and, in fact, outside the scope of this paper, which is how paleoenvironmental changes, such as shifts in climate, tectonics, and surface processes, influence these processes to shape biodiversity gradients. The paragraph attempting to link these hypotheses was potentially misleading, as we do not test these mechanisms independently. For these reasons, we chose to remove that paragraph in the revised version.

4. 67-76: It seems like the authors are trying to specify their modeling scope in this last paragraph but it is not 100% clear. I suggest being even more clear on exactly what processes and mechanisms are modeled and why. Also, they may consider merging this paragraph with the preceding one as I think it would improve the flow of the introduction.

Thank you for this comment. We merged the two paragraphs and inverted their order to improve readability. We now begin by explaining the need for integrating complex landscape dynamics beyond just climate and tectonics in investigating large scale biotic and abiotic interactions (Lines 42-46). We then more clearly state what we want to test in this study (Lines 46-56).

5. 111-115: Here the authors probably want to do a better job describing exactly what the biodiversity processes and mechanisms are. Maybe a conceptual figure or a table would help.

Thank you for your feedback. We provide more detailed descriptions of the mechanisms illustrated in each model to better distinguish them (Lines 84-93). A table (Table 2) describing the different modeled scenarios was already included (we consider it would be redundant with a conceptual figure), and this revised version has been moved to the beginning of the « Model specifics and parameters » section for better clarity and flow.

6. 117: It is unclear and a bit confusing to the reader, as it is not clear if these model specifics are included in all the scenarios mentioned above (also the ones that are "solely based on climate and tectonics").

Similarly to the comment above, we now more clearly acknowledge which parts of the model are common or dissimilar within each scenario (Lines 84-93). This structure is also described in the table 2.

7. 121-130: The way dispersal is framed as some cost is not intuitive to me as a first-time reader. I was hoping that the authors can frame this in a more intuitive way with more focus on actual dispersal and less on costs.

We recognize that this definition of dispersal can be counterintuitive, albeit technically useful. For a better understanding, we added a definition of what the cost represents (Lines 138-152), as well as an explanation of the default cost (Lines 141-144).

8. 132-136: I am sure the authors realize that this is an extremely simplified view of speciation. This is of course fine in a modelling study but the authors need to discuss the potential consequences of such simplifications for the results.

Great comment. We do realize that this view of speciation is simplified, and in the revised version, we have included a statement recognizing the potential consequences of this simplification (Lines 159-161).

9. 138-147: This section is described as if it was a Ornstin-Uhlenbeck process but it is really not as OU processes are commonly described as a continuous model. At the same time the authors mix in concepts like Brownian motion which is commonly completely undirected. This is confusing. I suggest that the authors revise this part such that it goes more in line with the standard terminology of directed stochastic models.

Interesting point, thank you. While we initially described this process using Brownian notion, our original description was misleading because a Brownian motion typically represents undirected evolution. Instead, our approach resembles a discrete-time , environmentally guided model in which trait values tend to move toward the prevailing local temperature. We have modified this description accordingly (Lines 163-175).

10. 226-239: For someone that is not an expert in these types of grid-based models this section becomes very dense. I suggest the authors try to revise this such that it becomes more accessible to non-experts.

This point is very understandable, and we have modified the explanations accordingly. However, since this part is technical, it is rather difficult to asses major changes without losing the reader in extensive explanations. We therefore decided to use more accessible wording for a broader audience, while avoiding lengthy explanations (Lines 110-124).

11. 253-261: Seems more appropriate to present this in the methods section. A reminder here in the results may be justified but should also be clearly described in the methods section.

Great point, thank you. We created a new section titled « Benchmarking and analyzing LDG through time », in which we present this methodological point (Lines 220-245).

12. 272-276: I may have missed somthing but I think that the authors fall short in actually "Understanding the LDG". Rather than concluding that biodiversity is correlated with precipitation (which is a trivial observation) they can actually disentangle some of the more interesting explanations that are based on dispersal, ecology, adaptation, and speciation. I thus call for a more indepth analyses of the different model scenarios Table 1 and how they preform and why.

We see your point ! Initially we wanted to describe the behavior of the models without revealing the final outcome of the paper by broadly presenting simple correlations. We have modified this section : comparing broad patterns (e.g. LDG arising from evolutionary responses to dynamic environmental conditions, with minimal impact from surface processes), emphasizing the dual role of biotic and abiotic factors (e.g. even with varied ecological settings, the richness gradients remain relatively stable, pointing to deeper evolutionary and macroenvironmental mechanisms as the primary drivers of LDG formation through time) (Lines 256-267).

13. 293: General: This whole section is very descriptive with very little explanations of why these patterns emerge. Given that the authors run different models (Table 1) they should be able to provide insights to the underpinnings of the observed patterns.

Great remark, this section was overly descriptive. In the revised version, this section is less descriptive, focusing better on explaining why these patterns emerge, without spoiling the deeper analyses and explanations that follow.

14. 376-377: This may be the most concerning result that comes with this study — the fact that different model scenarios give very similar outputs. It essentially means that the authors have limited information from the models that can help them disentangle the relative importance of different factors affecting the biodiversity patterns. The major goal of this paper thus seems to be out of scope for the particular model and model scenarios that were chosen by the authors.

We disagree, but understand that some clarification were required : these outcomes reveal the scale-dependent effects of surface processes, and we now more clearly elaborate in this (Lines 322-327). This observation indicates that—the broad pattern is set by the common attributes of all models : plate tectonics, that defines the land-sea mask and climate that set the geographical ranges of habitability, but that regional scales behave differently and respond to the specific parametrization of each models. In partirticular, physiographic diversity shapes diversification locally or within biogeographic regions but does not significantly contribute to broad LDG patterns. This is now made clear (Lines 322-325 and 328-329). Based on this understanding, we can then analyse the fundamental ways paleoenvironmental change shape biodiversity gradients through the effect on speciation, extinction and dispersal. Within the revised version, that goes straighter to the point, this should appear more clearly.

15. 431: In this section the authors provide some detail on potential drivers of biodiversity, but they are all more or less abiotic or environmental effects. Such explanations are quite different from the focus that was introduced in the introduction and early parts of the model presentation, i.e. dispersal, ecology, adaptation, and speciation. Thus it seems to me like the authors are missing their own mark that was set up in the introduction and early parts of the model description.

Thank you for this remark. We revised the Results section accordingly to better emphasize the scale-dependent influence of surface processes, highlighting more clearly the contrast between

large-scale patterns and the regional-scale patterns. As previously stated (Lines 322-326 and 328-329), broad patterns are set by the common attributes of all models (plate tectonics, defining the land-sea mask, and climate that set the geographical ranges of habitability), whereas regional patterns behave differently and respond to the specific parametrization of each models. We now restate this observation in this section (Lines 363-366). This allows us to explore more deeply how paleoenvironmental changes shape biodiversity gradients through their effects on speciation, extinction and dispersal (Lines 366-368), by more effectively linking biotic and abiotic interactions. These revisions follow the original focus stated in the introduction.

16. 522: Quite wordy section that does not provide a lot of insight. Repeating some of the key results and telling the reader that similar (or dissimilar)…

That is correct, we have revised this section to make it more concise and less redundant.

17. 568: Although the discussion about cradle and museum is interesting, I cannot help but ask the "why" question. I was hoping for more answers along the lines of the questions asked in the introduction of this paper.

This section now better address the initial question stated in the introduction, expressing the fundamental ways paleoenvironmental change shape broad diversification patterns, by stating the different influence of temporal, ecological and evolutionary mechanisms.

18. 621: In this section the authors are scratching the surface of what I am calling for above. I suggest the authors try to have such analyses of the comparison between model scenarios permeate the paper much better. This is where I see the real contribution from this modeling study.

The previous section and this section now do a better job describing the scale-dependent effects of surface processes on global-scale biodiversity patterns and better describe their combined influence during transient geological and climatic periods.

19. 699: This conclusions section does not bring much new or outstanding information. I suspect that this whole section can be deleted without loss of readability or clarity — given that the other parts of the discussion are tightened, that is.

We see your point and we have shortened the conclusion, pointing out aspects not previously discussed (e.g., importance of spatial and heterogeneity of landmasses, often overlooked in LDGs studies). However, as a conclusion should, we kept the key points of this study.

Reviewer 2 - General comments

1. Watch out for long sentences in abstract. Also, the abstract could benefit from ending with an outlook and future perspectives sentence.

We modified the abstract, following better the scope of the study. We also shorten the sentences.

2. 21-22: Definitions and distinctions between diversification and speciation probably needed here.

This is a great suggestion. However, as we mentioned before above (« Reviewer 1 – General comments »), we revised the Introduction to better narrow the focus of the paper and link it more clearly to our observations and discussion. The paragraph attempting to link these hypotheses was potentially misleading, as we do not test these mechanisms independently. Our focus is specifically on how paleoenvironmental changes shape biodiversity gradients through their effects on speciation, extinction and dispersal. As such, we removed the paragraphs listing biotic and

abiotic factors and linking different hypothesis underlying the LDG (Lines 7-9). In this revised version, we no longer reference speciation and diversification processes in shaping the LDG. For these reasons, we chose to remove this paragraph.

3. 49-50: A few follow-up sentences that provide explicit examples of mechanisms that have been modeled, and specific modeling studies and their results, may be good to add here, as it would help the reader understand what is meant and what possibilities these types of models provide.
We have added explicit examples, including the specific modeling framework and results that shape and build our understanding of the LDG (Lines 34-41).

4. 65: Even though the authors list key features of the model above, it may be worth being very clear — i.e., reminding the reader about what mechanisms they are focused on in this study.
Thank you for this comment. We merged the two paragraphs and inverted their order to improve readability. We now begin by explaining the need for integrating complex landscape dynamics beyond just climate and tectonics in investigating large scale biotic and abiotic interactions (Lines 42-46). We then more clearly state what we want to test in this study (Lines 46-56).

5. 81: “These dynamics” ,unclear — be specific.

We already stated what we are testing in this study in the introduction, since the paper is already quite long, we have removed this sentence.

6. 86-87: Not sure what this sentence is trying to say. I suggest either clarifying how the Game of Life model has inspired Gen3sis or deleting the sentence altogether.
Thank you for this comment. This reference highlighted the emergence and the beginnings of how simple models such as the Game of Life, have influenced the development of much more complex models by demonstrating the possibilities beyond this novel and simple approach. Since the paper is already quite long, we have removed this sentence (otherwise it would require more explanations).

7. 93: Not sure what this means — please clarify.
We modified the text accordingly by providing examples of what the boundary conditions are and what the behavior laws are referreing to (Lines 64-68).
8. 93: The reader is kept in the dark about what these biological functions and behavioral laws may be. I suggest that the authors provide a few examples here to relieve the reader’s frustration a bit.
As mentioned above, we modified the text accordingly by providing examples of what the boundary conditions are and what the behavior laws are referreing to (Lines 64-68).

9. 121: Please specify the temporal extent of such a time step.
Done « (here 1Ma) » (Line 139).
10. 121: Terminology used here implies that the reader should know what a "cost function" is and how it connects to dispersal. This is, however, not clear to me.

We grouped these two comments, and specified the temporal extent of the time step, and we also provided a clearer explanation of the «cost function» and how it relates to dispersal (Lines 138-152).

11. 132-136: As I am sure the authors realize, this is an extremely simplified view of speciation. This is of course fine in a modeling study like this, but the authors need to discuss the potential consequences of such simplifications for the results.
Great comment. We do realize that this view of speciation is simplified, and in the revised version, we have included a statement recognizing the potential consequences of this simplification (Lines 159-162).

12. 138-147: This section is described as if it were an Ornstein-Uhlenbeck process, but it is really not, as OU processes are commonly described as continuous models. At the same time, the authors mix in concepts like Brownian motion, which is commonly completely undirected. This is confusing. I suggest that the authors revise this part such that it aligns more closely with standard terminology for directed stochastic models.
Interesting point, thank you. While we initially described this process using Brownian motion, our original description was misleading because a Brownian motion typically represents undirected evolution. Instead, our approach resembles a discrete-time , environmentally guided model in which trait values tend to move toward the prevailing local temperature. We have modified this description accordingly (Lines 163-175).

13. 176-177: It may be worth describing why such an approach is conducted — i.e., a follow-up sentence describing how these 100 simulations were treated in downstream analyses.
Thank you for this suggestion. We have added a description of how the simulations were treated in downstream analyses (Lines 199-206).

14. 179: These 1 Myr time steps are huge in the context of dispersal and ecology. I strongly suggest that the authors discuss what consequences such long time steps, combined with the fact that different modeled processes act on very different time scales, have for the model behavior and output.
Great point to mention, thank you. We added a few sentences to explain the consequences of such a coarse time step and also clarified why this time step was chosen for the study (Lines 205-209).

15. 194-194: Should this not be part of the model specifics on dispersal presented above?
You are right, for better understanding, we combined the parameters with the model specifics and also created a section titled «Landscape modeling» that explains how the surface processes were modeled, placed before the model specifics.

16. 199: See comment above about OU process.
Great point, we revised this model description following the comment on OU process (Lines 202-209).

17. 221-224: Seems more like a discussion point than a statement in the methods section.
True, we removed this sentence, especially since it is discussed in the «Limitations» section.

18. Figure 4 would benefit from panel titles telling the reader which panel is showing which hemisphere results.
Thank you for this suggestion, we added panel titles telling the reader which panel is showing which hemisphere results.

19. 282: The authors fall short in justifying this claim. See my general call for in-depth analyses of the different model scenarios and how they perform — and why.
We understand your point. We added a section in the methodology that justify this claim called « Benchmarking and analyzing LDG through time » and in the « Present-day mammal diversity » section (Lines 256-267), and we aslo further developed the in-depth analyses of the different model scenarios in this section but also in the discussion section « Landscape dynamics and their legacy on biodiversity » (Lines 411-439).

20. 284-285: Not sure this is suitable for the results section — rather part of a discussion.
True, great remark. Since we already mentioned it in the «Limitations» section, we decided to remove this sentence.

21. 519-520: Yes, I agree that this is what the model can do and should do. But unfortunately, I do not think the authors utilize their model to its full potential. Rather than explaining the mechanisms underpinning LDGs, they resort to a very descriptive presentation of their results that essentially boils down to environmental and/or abiotic factors — despite the fact that they frame their study from several biotic perspectives in the introduction.
We see your point, thank you for your comment. We have revised this section (and globally the entire article along the same lines) so that it now better responds to the initial question stated in the introduction, exploring the interlinks between paleoenvironmental changes and broad biodiversification patterns via affecting dispersal pathways and diversifcation rates, which emerge from different ecological processes. Where stable climates promote species persistence and divergence; large landmasses enhance ecological opportunity and facilitate wide dispersal, while the combination of both landscape connectivity and mountainous regions foster isolation-driven speciation, especially at mid- to high latitudes, where climate instability limits diversification. Importantly, we reveal in this study the importance of spatial and heterogeneity of landmasses—particularly the North-South hemispherical asymmetry —in driving large-scale diversity patterns, an often overlooked aspect in LDG studies.
