# Supplementary material for: Deep time evolution of the Latitudinal Diversity Gradient: Insights from mechanistic models
Source: PLoS One. 2025 Sep 25;20(9):e0332766. doi: 10.1371/journal.pone.0332766 (PMC12463215; doi:10.1371/journal.pone.0332766)
Supplement: Attachment — Submitted filename: Response_to_Reviewers_auresp_2.pdf [file pone.0332766.s007.pdf]

Dear Dr Kiel,

We would like to thank you again for your careful evaluation of our manuscript and for forwarding us your helpfull comments. We are pleased that the remaining comments are minor and we have revised the manuscript accordingly.

In particular, we have modified the manuscript based on the following minor comments:

- abstract & throughout the manuscript: please use ’Cenozoic’ instead of ‘Tertiary’ – the latter term has been abandoned more than a decade ago.
  That is right, we have modified accordingly (abstract and lines 284 and 317).
- Line 5, perhaps ’recognized’ instead of documented.
  We replaced « documented » by « recognized » line 5.
- 21, ‘has been’
  We corrected « have been » by « has been » line 21.
- 34, permits
  We corrected « permit » by « permits » line 34.
- Fig. 1 caption: remove the last sentence, it just repeats the main text.
  Great comment, we removed the last sentence of the Fig. 1 caption.
- 87-88, please be clearer about M1s and M1d
  We clarified the differences between M1s and M1d as you pointed out, it was not very clear. To do so we stated : « Second scenario M1s integrates physical barriers (Φ) into speciation, with dispersal based on geographic distances (Δ). Dispersal in M1d is based on both geographic distances and physical barriers (Δ + Φ), with speciation depending solely on geographic distances (Δ). » (Lines 87-90).
- 440, amplified
  We corrected « amplify » by « amplified » line 442.
- 463, insert comma after ‘stability’
  We added a comma after « stability » line 465.
- 478, insert comma after ‘specialization’
  We added a comma after « specialization » line 480.
- 498-500, well, you did not investigate the time before the Cretaceous, so you cannot really point to a Cretaceous origin of the LDG.
  That is true, good point. To clarify this we now state « The main trend that our models show is that the LDG became established during the Cretaceous, and that the present-day LDG did so at around 50 Ma, then steepening, and taking its current form by approximately 35 Ma. » (Lines 500-502)
- 520-529, perhaps consider that the world entered an ice-house climate in the early Oligocene, with steepened temperature gradients from equator to poles.
  Great remark, we have added the idea that the Early Oligocene also marks the transition into icehouse climate, with steepened temperature gradients between the equator and poles, likely amplifying environmental heterogeneity and influencing diversification (Lines 522-533).

We believe these revisions fully address the minor comments and further improve the clarity and precision of the manuscript. We are grateful for the constructive feedback and for the opportunity to revise our work.

Thank you very much for your time and consideration.

Sincerely,

Manon Lorcery and co-authors
